# Effects of Exogenous SARS-CoV-2 S1 Protein and mRNA Vaccines on Mixed Neuronal–Glial Cell Cultures

**DOI:** 10.3390/medicina62010198

**Published:** 2026-01-17

**Authors:** Vytenis Markevičius, Eimina Dirvelytė-Valauskė, Urtė Neniškytė, Vilmantė Borutaitė

**Affiliations:** 1 Neuroscience Institute, Lithuanian University of Health Sciences, Eiveniu Str. 4, LT-50161 Kaunas, Lithuania; 2 Vilnius University Life Sciences Center-European Molecular Biology Laboratory (VU LSC-EMBL) Partnership Institute for Genome Editing Technologies, Life Sciences Center, Vilnius University, Sauletekio Str. 7, LT-10257 Vilnius, Lithuania; eimina.dirvelyte@gmc.vu.lt (E.D.-V.); urte.neniskyte@gmc.vu.lt (U.N.); 3 Institute of Bioscience, Life Sciences Center, Vilnius University, Sauletekio Str. 7, LT-10257 Vilnius, Lithuania

**Keywords:** SARS-CoV-2, neuroinflammation, mRNA vaccines, neurons, glial cells, microglia, inflammatory cytokines

## Abstract

*Background and Objectives*: SARS-CoV-2 produces potentially pathogenic molecules, such as single-stranded RNA and spike proteins, which can potentially activate microglial cells. In this study, we aimed to investigate whether SARS-CoV-2 spike protein S1 and mRNA vaccines can cause neurotoxicity directly or through microglial involvement. *Materials and Methods*: Primary cerebellar granule cell cultures isolated from Wistar rats and organotypic hippocampal slice cultures from transgenic C57BL/6J mice were used in the experiments. Imaging and quantitative analysis of cell viability, proliferation, and phagocytic activity were performed using light and fluorescence microscopy. *Results*: The exogenous SARS-CoV-2 S1 protein at 50 µg/mL concentration induced neuronal cell death in neuronal–glial co-cultures and stimulated microglial proliferation during the first 3 days of exposure without an effect on inflammatory cytokine secretion. Single application of Tozinameran/Riltozinameran and Original/Omicron BA. 4–5 vaccines did not affect neuronal viability and total neuronal number in cell co-cultures after 7 days of exposure. In contrast, three repeated treatments with mRNA vaccines at 6 ng/mL caused microglial proliferation without affecting microglial phagocytosis and TNF-α release. In organotypic brain slice cultures, only Tozinameran/Riltozinameran stimulated microglial cell proliferation in female brain slices, while male brain slices remained unaffected by both vaccines, indicating sex-dependent effects. *Conclusions*: The findings suggest that mRNA vaccines do not exert neurotoxic effects in primary neuronal–glial co-cultures, but induce microglial proliferation, particularly in female brains in the absence of inflammatory cytokine release. SARS-CoV-2 S1 protein at high concentrations directly induces neuronal death.

## 1. Introduction

The coronavirus disease (COVID-19) pandemic has increased interest in research on the cellular and molecular mechanisms of coronavirus-induced infection, as well as in the rapid development of mRNA vaccines. The severe acute respiratory syndrome coronavirus-2 (SARS-CoV-2) primarily acts on cells of the respiratory system; however, in some cases, systemic inflammation affecting multiple organs and tissues may develop [1]. There have been many reports that SARS-CoV-2 infection may result in various neurological complications, suggesting that it may also affect the central nervous system [2,3]. Post-mortem examination of COVID-19 patients’ brains had revealed gliosis and other markers of neuroinflammatory processes, including microglial activation [4]. However, it remains unclear whether this is caused by the direct action of the virus or its components on brain cells, or by a response to the activation of the peripheral immune system.

The SARS-CoV-2 S protein became the primary target for developing mRNA vaccines during the pandemic, as this protein binds to angiotensin-converting enzyme–2 (ACE2), allowing the entry of viral particles into cells [5], thus increasing viral replication and cellular damage, as was detected in lung tissues [6]. It has been shown that in the brain, SARS-CoV-2 can infect microglial cells and induce their death by apoptosis [7]. Additionally, it has been found that the SARS-CoV-2 S protein can bind to toll-like receptors and human ACE2 [5,8], resulting in the activation of the innate immune response cascade in microglia. SARS-CoV-2 S protein has been shown to increase the production of inflammatory cytokines IL-6, TNF-α, and IL-1β by BV-2 microglial cells [8], elevate TNF-α production in brain tissue [5], stimulate microglial oxygen consumption [9], cause activation of monoamine oxidase in neuroblastoma cells [10], stimulate purinergic signaling [11], and induce NLRP3 inflammasome activation [12]. However, whether previously reported effects of SARS-CoV-2 on microglia can also cause neuronal death remains unclear. Certain cytokines may upregulate microglial phagocytic activity, though consequent outcomes, such as neuronal phagocytosis by microglia, can be time-dependent [13,14]. Therefore, it is important to investigate whether prolonged exposure of co-cultures of brain cells to the SARS-CoV-2 S protein can influence neuronal loss and, if so, by which mechanism.

SARS-CoV-2 mRNA vaccines are based on lipid nanoparticle carriers that transfer mRNA of the SARS-CoV-2 S protein to cells for further protein translation [15]. In general, the safety and efficiency of all approved SARS-CoV-2 mRNA vaccines have been confirmed by many studies [16,17,18,19]. It has been shown that bivalent booster doses of SARS-CoV-2 mRNA vaccines can create better protection against different variants of the virus [20,21]. However, the dosing can influence SARS-CoV-2 S protein translation, inflammatory response, and immunogenicity, and cause differences in adverse side effects [22,23,24]. It has been reported that in some cases, application of SARS-CoV-2 mRNA vaccine booster doses may cause adverse side effects, including neurological deficits, such as Bell’s palsy and amyotrophic neuralgia [25], while [25] reported that neurological deficits usually occurred days to months after administering the vaccine, and it is unclear whether the outcomes were caused by the effects on neurons or glial/microglial cells. As the resident macrophages of the brain, microglia respond to infections and cytokines, contributing to inflammation, memory impairment, depression, synaptic plasticity, and neuronal loss [26], all of which can occur over long periods of time. Quick development and approval of SARS-CoV-2 mRNA vaccines evoked a lot of population studies [16,17,18,20,23,24,25], though the direct approach of mRNA vaccines on the brain is rarely investigated. Investigating mRNA vaccines on neuronal–glial cells excludes the possible effects of cytokines that cross the blood–brain barrier and activate microglia. Even though neuronal health is highly dependent on microglial activity, it has not yet been investigated whether mRNA vaccines can affect neuronal health directly or via microglial activity over longer periods of time.

Recently, it has been found that the SARS-CoV-2 spike protein influences microglial activity [5,8,9,10,11] and the SARS-CoV-2 mRNA vaccination has been shown to have neurological side effects, though their molecular mechanisms affecting neuronal health remain unknown. This study evaluates the effects of S1 from the SARS-CoV-2 S protein (receptor-binding domain) and two types of mRNA vaccines on primary neuronal–glial cell co-cultures. The aim was to determine whether SARS-CoV-2 S1 protein and mRNA vaccines can directly affect neuronal viability or cause microglial activation, leading to neuronal loss during prolonged (3–7 days) exposure.

## 2. Materials and Methods

### 2.1. Materials

Dulbecco’s Modified Eagle Medium (DMEM, high glucose, GlutaMAX, Cat# 61965026, Gibco-Invitrogen, Paisley, UK); Dulbecco’s Modified Eagle Medium (DMEM High Glucose w/L-Glutamine w/o Sodium Pyruvate—L0102, Biowest, Nuaillé, France); COVID-19 mRNA Vaccine (nucleoside-modified) 15/15 µg/dose Tozinameran/Riltozinameran (Comirnaty, EU/1/20/1528/XXX, PAA189718, EXP date: April 2023 BioNTech Manufacturing GmbH, Mainz, Germany); COVID-19 mRNA Vaccine (nucleoside-modified) 15/15 µg/dose Original/Omicron BA.4-5 (Comirnaty, EU/1/20/1528/XXX, PAA194702, EXP date: July 2023 BioNTech Manufacturing GmbH, Mainz, Germany); Rotenone (Cat# R8875, Sigma-Aldrich, St. Louis, MO, USA); isolectin GS-IB4 Alexa Fluor 488 Conjugate (Cat# I21411, South San Francisco, CA, USA); Hoechst 33342 (Sigma-Aldrich, St. Louis, MO, USA); Hanks’ Balanced Salt Solution (HBSS; Gibco; Thermofisher Cat# 14025092, Paisley, UK); SARS-CoV-2 S1 recombinant protein (Thermofisher-Invitrogen; Cat# RP87681, Waltham, MA, USA); β-nicotinamide adenine dinucleotide reduced disodium salt hydrate (NADH) (Sigma-Aldrich, St. Louis, MO, USA); coenzyme Q_2_ (2,3-dimethoxy-5-methyl-6-geranyl-1,4-benzoquinone; Sigma-Aldrich, St. Louis, MO, USA); horseradish peroxidase (Type I) HRP (Sigma-Aldrich, St. Louis, MO, USA) lot # SLBF8265V; and Poli-L-lysine (Cultrex, Minneapolis, MN, USA) Cat# 3438-100-01; Versene (1×) (Gibco, Cat# 15-040-066, Paisley, UK).

### 2.2. Animals and Ethical Approval

All experimental research that included rats was undertaken in accordance with the EU Directive 2010/63/EU for animal experiments and the Republic of Lithuania law on the care, keeping, and use of experimental animals (approved by Lithuanian State Food and Veterinary Service, ethical approval No. G2-267, 15 January 2024). The rat pups were bred and maintained at the Lithuanian University of Health Sciences animal house under controlled conditions. The pups were decapitated.

Thy1::EGFP; Cx3cr1::CreER; RC::LSL-tdTomato triple transgenic mice (C57BL/6J con-genic background) were obtained from colonies at the Life Sciences Center, Vilnius University. Murine genotyping and colony maintenance was previously described [27]. Animal studies were approved by the Lithuanian State Food and Veterinary Service (ethical approval No. B6-(1.9.)-2653, 27 November 2020). All mice were bred and kept at the animal facility at the Life Sciences Center of Vilnius University.

### 2.3. Primary Rat CGCs

Cerebellar granule cell cultures (CGC) were prepared from cerebella of 5–7-day-old Wistar rats of both sexes as previously described [28]. Cell cultures were isolated by dismembering the rat cerebellum in Versene (1×) (0.48 mM, in PBS with 0.2 g/L EDTA(Na4)). Cells were suspended in DMEM GlutaMAX high glucose containing 1% penicillin/streptomycin, 5% fetal bovine serum, 5% horse serum, 20 mM KCl, 13 mM glucose [29]. Cells were plated on poly-L-lysine (1 μg/mL) precoated 96-well and 48-well plates at 0.65 × 10^6^ cells/mL and 10^6^ cells/mL densities, respectively. For the 96-well Half Area High Content Imaging Glass Bottom Microplate (CORNING, 4580, Corning, NY, USA), we used a 0.4 × 10^6^ cells/mL density. Cells were grown at 37 °C in an incubator with 5% CO_2_ for 5–7 days before treatment.

### 2.4. Primary Glial and Pure Microglial Cell Cultures

Primary glial cultures were prepared from 5- to 7-day old Wistar rat cerebral cortex as previously described [30]. After isolation, the cerebral cortex was placed in a Petri dish with 4 °C HBSS and 1% penicillin/streptomycin. Blood vessels were removed with tweezers. Then, the cerebral cortex was dismembered in Versene (1×) (0.48 mM, in PBS with 0.2 g/L EDTA(Na4)) and suspended in DMEM GlutaMAX high glucose, without pyruvate, and supplemented with 20% fetal bovine serum and 1% penicillin/streptomycin. Cells were plated in T75 flasks precoated with 0.5 μg/mL poly-L-lysine. After 24 h, the whole cell medium was changed. Cell cultures were grown for 7–10 days in DMEM GlutaMAX high glucose, without pyruvate, supplemented with 10% fetal bovine serum and 1% penicillin/streptomycin, changing half of the medium every 3 days in an incubator at 37 °C with 5% CO_2_. For later use (See Section 2.9), glial cells were detached by shaking flasks mechanically for 10–15 min.

Isolation of pure microglial cells from the rest of glial cultures was made from 7- to 14-day old glial cultures. Microglial cells were separated by shaking the flasks mechanically for 6–7 min, then centrifuging at 270× *g* for 5 min and resuspending at 10^5^ cells/mL density in the growth medium. Cells were seeded in 48-well plates precoated with 5 μg/mL poly-L-lysine and allowed to attach to the surface for 24 h before applying experimental treatments.

### 2.5. Organotypic Hippocampal Slice Cultures

Organotypic hippocampal slice cultures were prepared as previously described [27,31]. Briefly, 3-day-old pups of Thy1::EGFP; Cx3cr1::CreER; RC::LSL-tdTomato mice were decapitated, and hippocampi were dissected out in a Petri dish filled with an ice-cold dissection medium (100 U/mL penicillin, 100 µg/mL streptomycin, 15 mM HEPES, 0.5% glucose in HBSS). Hippocampi were sliced at a thickness of 300 μm using a McIlwain tissue chopper. The slices were placed onto PTFE cell culture inserts (0.4 μm pore size, 30 mm diameter; Merck Millipore) in a 6-well plate filled with prewarmed maintaining medium (25% 1× BME, 25% horse serum, 5% 10× MEM, 100 U/mL penicillin, 100 µg/mL streptomycin, 2 mM GlutaMAX, 0.65% glucose, 9 mM sodium bicarbonate in ddH_2_O). Cultures were maintained in a 5% CO_2_ incubator at 37 °C. The medium was changed 24 h after the plating and every 2–3 days during culture maintenance. Slices were cultivated on the inserts for 14 days before experiments were performed, as described in the following sections. After vaccine experiments, organotypic hippocampal slice cultures were fixed with 4% paraformaldehyde (PFA) in PBS for 30 min. at room temperature. Residual PFA was removed by rinsing with PBS and quenched with 30 mM glycine in PBS twice for 15 min. at room temperature. After quenching, the cultures were washed twice with PBS.

### 2.6. Cell Viability

Cell viability was evaluated with a fluorescence microscope (Olympus IX71, Tokyo, Japan) using 20× magnification and staining with 40 µg/mL Hoechst 33342, 10 µg/mL propidium iodide, and 7 ng/mL isolectin GS IB4 AlexaFluor 488 [29]. Propidium iodide-positive cells were counted as necrotic, while apoptosis was assessed by Hoechst-marked condensed/fragmented nuclei [28,30]. Isolectin was used to identify microglial cells in mixed neuronal–glial cultures. Cells were counted in 5 randomly chosen microscopic fields per well.

### 2.7. Microglial Phagocytic Activity

Microglial phagocytic activity was assessed using 2 µm fluorescent latex beads in pure microglial cultures (see Section 2.4). Briefly, after vaccine treatment, microglial cell monocultures were supplemented with 0.005% (*w*/*v*) latex beads, carboxylate-modified polystyrene, and fluorescent red (Sigma-Aldrich, St. Louis, MO, USA) for 2 h. Microglial cells were stained with isolectin GS-IB4 Alexa Fluor 488 conjugate and Hoechst 33342. Afterwards, the treatment cells were rewashed 3 times with PBS, gently rotating the plate for 1 min each time to fully remove non-engulfed beads, and fixed with 4% paraformaldehyde for 15 min. After fixation, cells were washed with PBS 1 time and kept in PBS for the rest of the imaging process. Cells were assessed using a fluorescence microscope in 5 randomly chosen 20× magnification fields.

### 2.8. Vaccine Maintenance and Preparation for Cell Culture Treatments

Vaccines were provided by the Lithuanian Ministry of Health, in cooperation with the Neuroscience Institute of the Lithuanian University of Health Sciences. Vaccines were transported on dry ice and stored at −80 °C for later use. Each vial contained 100 µg/mL (30 µg per 300 µL dose) of lipid nanoparticles with SARS-CoV-2 spike mRNA.

Vaccines were thawed at room temperature for 30 min before the experiment. For each independent experiment, a new vial of mRNA vaccine was used. Neuronal–glial co-cultures were treated with singular 1–100 ng/mL doses of the vaccines for 3- and 7-day periods to assess cell viability, microglial proliferation, and neuronal density. Concentrations were chosen according to a similar type of vaccines used for immunization research [17,20,23,24] (10–250 µg of vaccine per person). We standardized vaccine doses ranging from 10 to 250 µg to a blood volume of 5 L to more closely mimic physiological conditions.

In another series of experiments, a 6 ng/mL dose of the vaccine, standardized to the single dose used for adult human immunization (30 µg of vaccine per 5L of blood), was administered. Neuronal–glial co-cultures, organotypic slices, and primary microglial cultures were treated with a standard 6 ng/mL dose of SARS-CoV-2 mRNA T/R and O/OBA vaccines every 24 h for 3 days (final conc. 18 ng/mL), then the cells were left to incubate for 4 more days.

During each independent experiment, experimental conditions were repeated in two separate wells.

### 2.9. Mitochondrial Complex I Activity Assay

For mitochondrial complex I activity, we used 7–14-day old glial cultures (see Section 2.4). A total of 1.5 × 10^6^ of glial cells were seeded in T25 flasks precoated with poly-L-lysine (1:20). After 7-day incubation with T/R and O/OBA vaccines, cells were scraped and centrifuged at 1000× *g* for 5 min. The cell pellet was resuspended in 200 µL of distilled water. The cell suspension was exposed to 3 freezing/thawing cycles and sonicated for 2 min at 50 Hz in an ultrasound bath without heating. The sample protein concentration was evaluated using the Biuret method. NADH oxidation rate was measured with a NanoPhotometer™ Pearl (IMPLEN; Munich, Germany) UV–VIS spectrophotometer. A total of 0.45 mg of sample protein was added to a cuvette at room temperature containing 1 mL of 10 mM K_2_HPO_4_/KH_2_PO_4_ (pH 7.5) and mitochondrial complex I substrate—400 µM β-nicotinamide adenine dinucleotide reduced disodium salt hydrate (NADH). NADH oxidation by mitochondrial complex I was initiated by adding coenzyme Q2 (CoQ2) to a final concentration of 100 µM. The NADH oxidation curve was measured at 340 nm absorption wavelength. Firstly, the NADH oxidation curve was recorded for 2 min, then the complex I inhibitor rotenone at 5 µM was added directly to the sample, and the oxidation curve was followed for 2 more minutes. The rate of NADH oxidation was calculated using the NADH molar extinction coefficient of 6.81 mM^−1^ cm^−1^, as previously described [32,33].

### 2.10. Hydrogen Peroxide Measurements

CGCs were seeded in 96-well Half Area Black/Clear Flat Bottom High Content Imaging Glass Bottom Microplates (Corning, Corning, NY, USA). Cells were treated with a 3 × 6 ng/mL (18 ng/mL final) concentration of T/R and O/OBA mRNA vaccines for 7 days. H_2_O_2_ production rate was measured using an AmplexRed reagent (ThermoFisher, Waltham, MA, USA) assay. For measurements, growth media were replaced with 50 µL of HBSS containing 0.03 µg/mL Horseradish peroxidase and 15 µM AmplexRed reagent. Measurements were made with a Fluoroskan Ascent (ThermoFisher, Waltham, MA, USA) plate fluorimeter using 544 nm excitation and 590 nm emission wavelengths, scanning every 5 min for 1 h. After measurements, cells were stained with 4 µg/mL Hoechst33342, washed with HBSS, and visualized in each well using a EVOS M7000 imaging system (ThermoFisher, Waltham, MA, USA) under 20× magnification. Cells were counted in a 50% area of each well using the EVOS^TM^ analysis (ThermoFisher, Waltham, MA, USA) system counting only Hoechst-stained nuclei. The calculated rate of H_2_O_2_ production was normalized to 10^4^ cells per well.

### 2.11. SPIM/LSFM Imaging

Imaging of fixed microglia and neurons in murine hippocampal slice cultures was performed on a Light-Sheet Z1 microscope (Carl Zeiss Microscopy Germany GmbH, Oberkochen, Germany), as previously described [34]. Briefly, the membrane with the fixed murine hippocampal slices was cut around the sample using a scalpel. Then, using microscopic tweezers, each sample was mounted in a glass capillary (inner ⌀ 0.68 mm) with a plunger and filled with 1% low-gelling temperature agarose (Sigma-Aldrich, St. Louis, MO, USA) in PBS at 37 °C. The capillary was gently rolled for 1–2 min in a horizontal position for the sample to remain in the center of the capillary, while agarose reaches its polymerization temperature. The capillaries were mounted in an imaging chamber, filled with PBS at 25 °C, and prewarmed for 1 h before imaging. Agarose gel was gently pushed out to fully expose the sample to the imaging area. Imaging was performed only in those slice regions that consisted of largest number of neurons in the field. For this, we used dual-side illumination. Focus stacking (Z-stacking) was performed for 15–20 min using 0.5 zoom with 40×/1.0 W Plan-APO objective (ZEISS), set to 0.215 µm step size and 200 µm depth. Image acquisition was performed using a 488 laser with 525–565 nm band-pass emission filter for GFP (Neurons) and a 561 nm laser with 580 nm Longpass filter for tdTomato and iRFP (Microglia). To fuse the z-stacks of two-side illumination, we performed DFT fusion (Discrete Fourier Transform Fusion). Fused z-stacks were processed into maximum intensity projections for cell counting and statistical evaluation. All image processing was performed with ZEN 3.4 (blue edition, Carl Zeiss Microscopy Germany GmbH, Oberkochen, Germany).

### 2.12. TNF-α and IL-6 Measurements

Cytokines were measured with rat IL-6 (Abbexa; abx050126, Cambridge, UK) and rat TNF-α (Abbexa; abx256060, Cambridge, UK) ELISA kits. After incubation with vaccines and SARS-CoV-2 S1 protein, cell culture media were collected and centrifuged at 1000× *g* for 10 min and stored at −20 °C. For TNF-α and IL-6 measurements, 100 µL of collected cell culture media were used. All procedures were carried out according to the manufacturers’ protocols.

### 2.13. Statistics

Statistical analysis was performed using GraphPad Prism (version 10.5.0) software. Data normality was determined by the Shapiro–Wilk test. Homogeneity of variances was tested with the Brown–Forsythe test. The S1 protein effect was measured using two-way ANOVA with uncorrected Fisher’s least significant difference test (LSD). For mRNA vaccine effects, ordinary one-way ANOVA with Tukey’s, and for nonparametric data, we used Kruskal–Wallis with uncorrected Dunn’s multiple comparison test. Hippocampal slice data statistical analysis was performed by two-way ANOVA using Tukey’s multiple comparisons. All statistical analyses were performed at the 95% confidence interval. Data was collected from at least 3 independent cell cultures.

### 2.14. SARS-CoV-2 S1 Preparation for Cell Culture Treatments

A total of 100 µg of SARS-CoV-2 S1 recombinant protein (Thermofisher-Invitrogen; Cat# RP87681, Waltham, MA, USA) stock solution was prepared in 200 µL sterile PBS (final concentration 0,5 mg/mL). The concentrations of SARS-CoV-2 S1 protein (1–10 μg/mL) were selected based on previously published studies demonstrating biological activity within this range [11,12,35]. Additionally, we added a previously not investigated 50 μg/mL concentration.

## 3. Results

### 3.1. The Effect of SARS-CoV-2 Spike S1 Protein on CGCs

**Figure 1:**
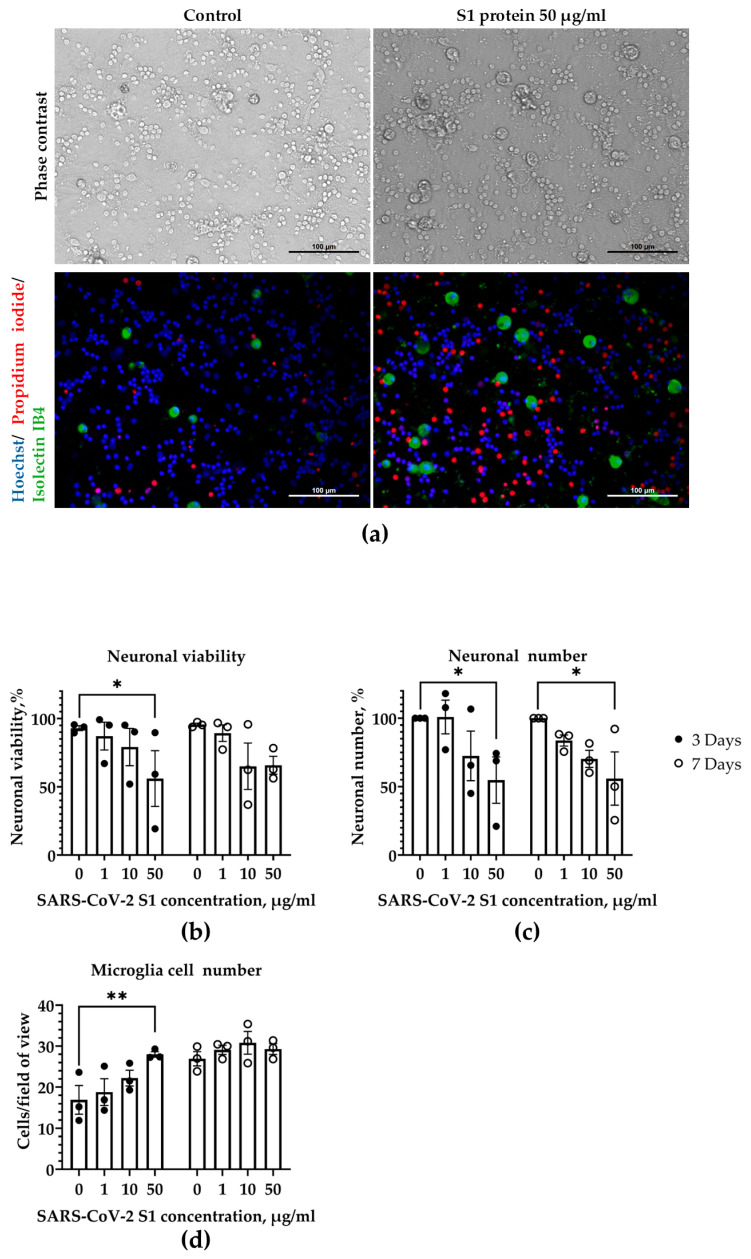
The effects of SARS-CoV-2 spike S1 protein on CGCs after 3- and 7-day treatment. (**a**) Representative images of control and S1 protein (50 µg/mL)-treated cultures after 3 days. CGCs were incubated in the presence of S1 protein at concentrations indicated in the legend. Cell nuclei were stained with Hoechst 33342 (blue for all nuclei) and propidium iodide (red indicates nuclei in necrotic cells). Neuronal cells were identified based on their distinct morphology (in Phase contrast). Neurons stained homogeneously with Hoechst 33342 were considered viable, while displaying condensed nuclei—apoptotic. Microglial cells were labeled with isolectin-IB4-AlexaFluor488 (green). Cells were counted in at least 5 random microscopic fields in each well. Scale bar represents 100 µm. (**b**) The percentage of viable neurons per microscopic field in the treatment groups; (**c**) the number of neuronal cells per microscopic field expressed as a percentage of neuronal cell numbers in the respective control group; (**d**) microglial cell number displayed as cells/20× microscopic field. Data are presented as means ± SEM of 3 independent experiments on separate cell cultures. * *p* < 0.05; ** *p* < 0.01, using LSD multiple comparison test.

To investigate whether SARS-CoV-2 spike S1 protein exerts an effect on mixed neuronal–glial cultures over time, we used CGCs that consisted of neurons, microglia and astrocytes. We found that after treatment with 1–10 µg/mL SARS-CoV-2 S1, neuronal viability after 3-day incubation was not significantly different from the viability of neurons in control cultures (Figure 1b). In contrast, a significant decrease in neuronal viability by 44% was observed after 3-day treatment with 50 µg/mL S1 protein (two-way ANOVA of Treatment effect F _(3,16)_ = 3.41, *p* = 0.04) and remained at that level after 7-day treatment. The SARS-CoV-2 S1-induced decrease in viability was due to neuronal necrosis, as evidenced by the presence of a high number of neurons with propidium iodide-positive nuclei in CGCs after treatment with 50 µg/mL S1 protein (Figure 1a). The total number of neurons in CGCs was not significantly affected by the treatment with 1–10 µg/mL S1 protein for 3 and 7 days, while at 50 µg/mL concentration, the number decreased by about 45% after 3-day incubation and remained at a similar level after 7-day incubation (Figure 1c).

While 1–10 µg/mL S1 had no effect on microglial proliferation (Figure 1d), the quantitative analysis demonstrated a significant effect of 50 µg/mL S1 on the total microglial cell numbers: 28 ± 1 cells/field (F _(3,16)_, *p* = 0.04) after 3-day treatment compared to 17 ± 3 cells/field in the untreated control. After 7-day treatment with S1 protein, there was no significant difference in total microglial counts between control samples and S1-treated groups in a range of 1–50 µg/mL concentrations (Figure 1d).

To determine whether SARS-CoV-2 S1 protein affects cytokine secretion, we measured TNF-α and IL-6 levels in cell culture media after 3- and 7-day exposure to 50 µg/mL S1 protein. The exposure to the S1 protein did not change TNFα secretion after 3 days (26 ± 1 pg/mL) nor after 7 days (25 ± 3 pg/mL), compared to a 7-day untreated control group (21 ± 1 pg/mL), while IL-6 was not detectable in CGCs at all.

Altogether, these data suggest that a single application of SARS-CoV-2 S1 protein at a high concentration of 50 µg/mL induces neuronal necrosis and stimulates microglial proliferation in CGCs only for a short 3-day period.

### 3.2. The Effects of mRNA Vaccines on CGCs

**Figure 2:**
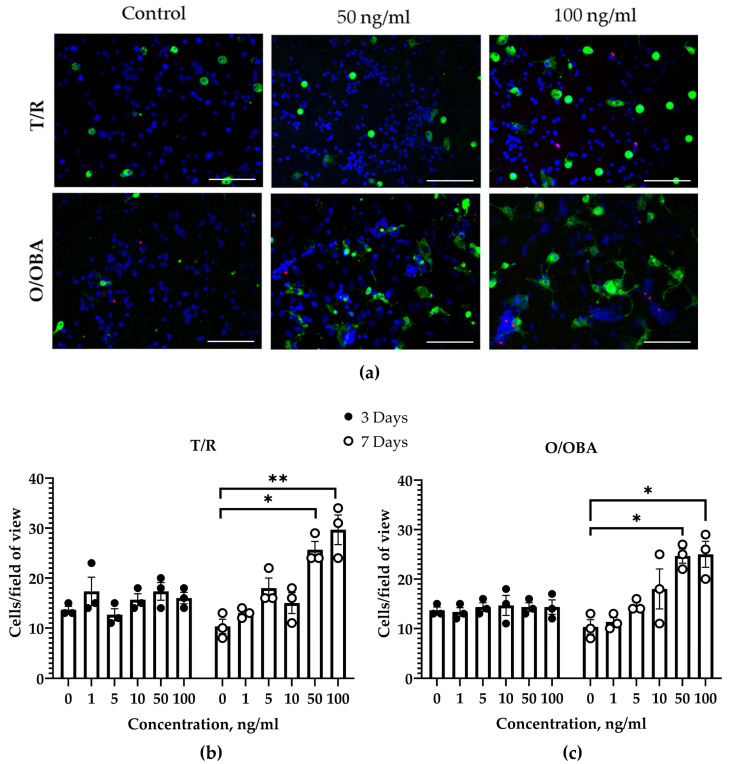
The effects of SARS-CoV-2 mRNA vaccines on microglial proliferation in CGC co-cultures after 3- and 7-day treatment. (**a**) In the representative images, microglial cells were distinguished according to fluorescent IB4-AlexaFluor488 (green) dye. Neurons with nuclei homogeneously stained with Hoechst 33342 (blue) were considered viable, whereas neurons with propidium iodide-positive nuclei (red) were counted as necrotic. No condensed and fragmented nuclei were detected, suggesting there were no apoptotic cells. Scale bar represents 100 µm (**b**) T/R vaccine effect on microglial proliferation; (**c**) O/OBA vaccine effect on microglial proliferation. Single doses of vaccines at indicated concentrations were added, and CGCs were incubated for 3 and 7 days. Microglial cells were identified after labeling with isolectin-IB4-AlexaFluor488. Graphs represent the number of microglia cells/20× magnification microscopic field. Untreated control CGCs after 3-day incubation consisted of 92 ± 2% neurons, 6 ± 2% microglia, and 2 ± 1% astrocytes, while after 7 days of incubation: 89 ± 0.5% neurons, 6 ± 1% microglia, and 5 ± 3% astrocytes. Data are presented as means ± SEM of 3 independent experiments on separate cell cultures. * *p* < 0.05; ** *p* < 0.01 using Dunn’s multiple comparison test.

Next, we investigated whether T/R and O/OBA mRNA vaccines can exert direct effects on CGCs. We found that both vaccines in the range of 1–100 ng/mL concentrations had no significant effect on neuronal viability after 3–7-day incubation: cell viability was 95–98% in all experimental groups (Figure S1a,b). The total number of neuronal cells was also similar in the untreated control and the vaccine-treated groups after 3 and 7 days (Figure S1c,d). T/R and O/OBA vaccines had no effect on microglial proliferation during the 3-day treatment (Figure 2b,c); however, after 7-day incubation, an almost 2-fold increase in the total microglial cell count per microscopic field was observed in cultures treated with vaccines: 26 ± 2 cells/field at 50 ng/mL T/R and 30 ± 3 cells/field at 100 ng/mL T/R; with O/OBA—25 ± 2 cells/field at 50 ng/mL and 27 ± 1 cells/field at 100 ng/mL compared to 12 ± 1 cells/field in the untreated control (Figure 2).

**Figure 3:**
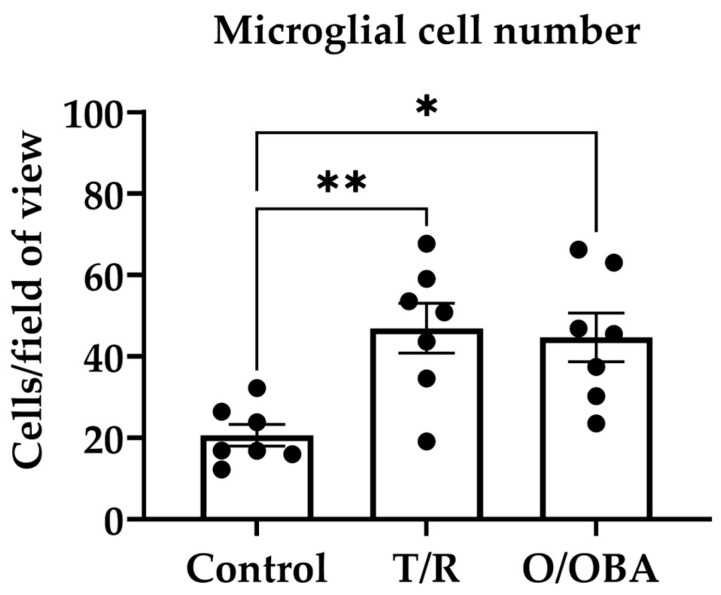
The effects of repeated treatment with SARS-CoV-2 mRNA T/R and O/OBA vaccines on microglial proliferation in mixed neuronal–glial co-cultures after a 7-day incubation. A total of 6 ng/mL was added to the cell culture every 24 h for 3 days. On the seventh day of the experiment, neuronal viability and microglial proliferation were evaluated. The average numbers of microglial cells per microscopic field are presented. Untreated control CGCs consisted of 90 ± 2% neurons, 8 ± 2% microglia, and 2 ± 1% astrocytes. Data are presented as means ± SEM of 7 independent experiments on separate cell cultures. * *p* < 0.05; ** *p* < 0.01—statistically significant effect compared to the control group using Tukey’s multiple comparison test, all data presented as means ± SEM.

During immunization against SARS-CoV-2, vaccination is repeated at least two times [17]. Therefore, we investigated whether three repeated applications of a standard (6 ng/mL) dose of T/R and O/OBA mRNA vaccines can affect CGCs during 7-day treatment. We found that repeated additions of T/R and O/OBA vaccines doses had no effect on neuronal viability or the total number of neurons in CGCs (Figure S2). In contrast, the analysis of microglial cell numbers in CGCs revealed that both vaccines increased the total microglial cell number by about twofold compared to the untreated control group: the total microglial cell count was 22 ± 3 cells/field in control, 45 ± 6 cells/field in T/R- and 44 ± 6 cells/field in O/OBA-treated cultures (one-way ANOVA (F _(2,19)_ = 1.03, *p* < 0.01), as presented in Figure 3. These data suggest that repeated application of standard doses of vaccines over the 7-day period results in stimulation of microglial proliferation without an effect on neuronal viability.

We also measured TNF-α levels in cell culture media after repeated treatments with T/R and O/OBA vaccines. We found that after the 7-day incubation of CGCs with repeated doses of mRNA vaccines, TNF-α was below detection level in all groups (below 15.6 pg/mL).

It is known that H_2_O_2_ is one of the signals for cell proliferation, and suppression of mitochondrial respiratory complex I activity may serve as a source of intracellular H_2_O_2_. Thus, we investigated whether mRNA vaccines can elevate the production of cellular H_2_O_2_ after the 7-day incubation of CGCs with the repeated application of 6 ng/mL vaccine doses, and whether this can be related to the inhibition of mitochondrial respiratory complex I. We found that the H_2_O_2_ production rate in CGCs did not significantly change after the repeated application of vaccines: it was 16 ± 1 nmol H_2_O_2_/min in T/R- and 18 ± 2 nmol H_2_O_2_/min in O/OBA-treated cultures compared to 13 ± 1 nmol H_2_O_2_/min in the control group. In accordance, we found that vaccines had no significant effect on mitochondrial respiratory complex I activity in glial cultures: 149 ± 31 nmol/min/mg protein in control cultures, 175 ± 22 nmol/min/mg protein in T/R-treated cultures, 156 ± 18 nmol/min/mg protein in O/OBA-treated cultures.

### 3.3. The Effects of mRNA Vaccines on Microglial Monocultures

**Figure 4:**
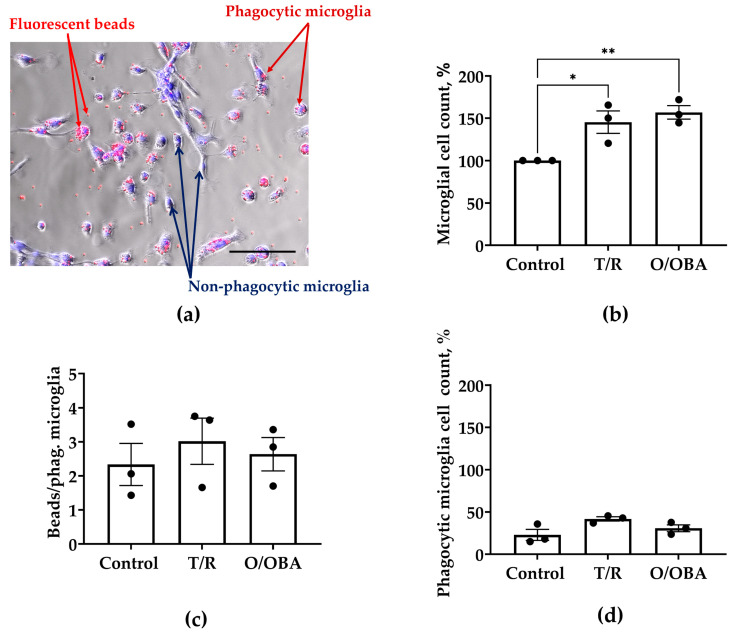
The effect of repeated treatment with T/R and O/OBA vaccines on microglial monocultures. (**a**) Representative images of microglial engulfment of carboxylate-modified fluorescent latex beads (red). Microglial nuclei stained with Hoechst 333342 dye (blue). Scale bar represents 50 µm; (**b**) effect of T/R and O/OBA on microglial cell numbers; (**c**) number of engulfed beads per phagocytic microglia; (**d**) number of phagocytic microglia presented as percentage of microglia that engulfed 1 or more fluorescent beads. Repeated doses of vaccines were applied to microglial cell cultures, and assessment was performed 7 days after the treatment as described in Methods. Microglial cell number is expressed as a percentage of the total number of microglia in the respective control group. Phagocytic microglial cell number is expressed as a percentage of total microglia that engulf 1 or more beads per total number of microglia in the treatment group. Data information: * *p* < 0.05; ** *p* < 0.01 statistically significant effects compared to the control group using Tukey’s multiple comparison test, all data presented as means ± SEM.

In the next series of experiments, we sought to determine whether mRNA vaccines directly affect microglial cells. To do this, we used microglial cell monocultures that received repeated treatment with T/R and O/OBA vaccines. As shown in Figure 4b, after 7-day incubation, microglial cell numbers increased by 48 ±11% in T/R-treated and by 57 ± 8% in O/OBA-treated compared to the control monocultures (F _(2,6)_ = 11.40, *p* < 0.01).

Activated, proliferating microglia may exhibit increased phagocytic capacity. We evaluated this by quantifying the number of fluorescent latex beads engulfed by microglia (Figure 4a). The percentage of microglial cells with beads inside was not significantly different in the control and treated with vaccines cultures: 23 ± 6% phagocytic microglia in the control group compared to 42 ± 3% in T/R-treated and 31 ± 4% in O/OBA-treated cultures (Figure 4d). The capacity of microglia to engulf beads was also unchanged after repeated treatment with vaccines: 2 ± 1 engulfed beads/cell in the control group, compared to 3 ± 1 beads/cell in T/R- and 3 ± 1 beads/cell in O/OBA-treated cultures.

These findings suggest that mRNA vaccines directly stimulate microglial proliferation, whereas microglial phagocytic activity remains unchanged.

### 3.4. The Effect of mRNA Vaccine Treatment on Murine Hippocampal Slice Cultures

**Figure 5:**
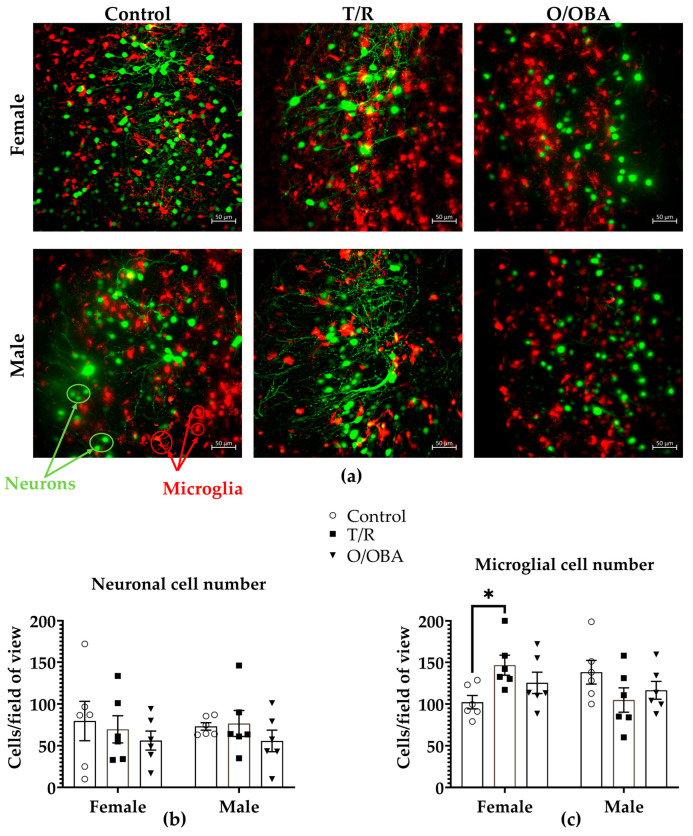
Organotypic murine brain slices after repeated application of T/R and O/OBA vaccines. (**a**) SPIM/LSFM images of murine hippocampal slice cultures after repeated application of mRNA vaccines. Images at 20× magnification, a microscopic field, represent a projection of a 200 µm depth z-stack (focus-stack). In the images, green color indicates GFP-labeled neurons, while red color indicates RFP-labeled microglia. Scale bar represents 50 µm. (**b**) This image shows the total neuronal cell count per 20× microscopic field; (**c**) shows the total microglial cell/20× microscopic field. All data presented as means ± SEM, where each dot represents an individual animal (*n* = 6). * Statistically significant effect compared to respective control (*p* < 0.05), according to ordinary two-way ANOVA design with Tukey’s multiple comparison tests.

In further experiments, we tested the effects of vaccines on neuronal viability and microglial proliferation in a more physiological model—murine hippocampal slice cultures that preserve the 3D architecture of brain tissue. We analyzed LFSM data from over 118 murine hippocampal brain slices from six female and six male mice to measure microglial proliferation and neuronal density. To observe neuronal and microglial cells, we used transgenic mice expressing fluorescent markers green fluorescent protein (GFP) in neurons and tdTomato in microglia, as shown in LSFM images (Figure 5a).

Firstly, the quantitative analysis showed that the treatment with repeated applications of T/R and O/OBA vaccines had no significant effect on the total neuronal cell count both in female and male groups compared to their respective controls (total neuronal cell count ± SEM; females: control 80 ± 24, T/R 70 ± 16, O/OBA 56 ± 11; males: control 73 ± 4, T/R 76 ± 16, O/OBA 56 ± 13) (Figure 5b). We found that the total microglial number significantly increased in T/R-treated group (147 ± 12 cells), compared to the female control (102 ± 8 cells), as presented in Figure 5c. Two-Way ANOVA analysis showed that there was a significant interaction effect, since vaccines had different effects on microglial proliferation between females and males (F _(2,30)_ = 5.062, *p* = 0.013), as there was a difference between the effects of vaccine treatments in different genders. In contrast, the gender (F _(1,53)_ = 0.005, *p* = 0.946) and treatment (F _(2,53)_ = 0.763, *p* = 0.471) overall factors were insignificant.

## 4. Discussion

In this study, we demonstrate that SARS-CoV-2 S1 protein added directly to the brain cell cultures can reduce neuronal viability but only at a relatively high 50 µg/mL concentration, which also acutely stimulated microglial proliferation for a short, 3-day period. The reasons why the stimulation of microglia was transient are not entirely clear but may be due to the clearance of the protein from cells or could be related to the development of cellular adaptive responses. It has been previously reported that the S1 protein can exhibit proinflammatory properties in vitro [8,36] and that S1 may function independently of viral infection and induce neuroinflammatory responses in rats [5,7,37]. In principle, our findings on S1 protein effects on CGCs are in line with these previous reports, though we did not find the production of TNFα or IL-6 in neuronal–glial co-cultures. It is possible that such differences observed in the present and previous studies were due to differences in cell cultures: pure microglial cultures were used in previous studies that found an elevation in proinflammatory cytokines [5,7,8], whereas our experiments were performed on mixed neuronal–glial co-cultures, which contained less than 10% glial cells.

S1 protein at a relatively high, 50 µg/mL concentration was found to induce neuronal necrosis, accompanied by a reduction in neuronal cell numbers in CGCs, which is most likely related to the removal of dead cells by microglia. S1-induced direct neuronal death can be caused by a variety of pathways. It has been reported that recombinant S1 protein can be internalized via the surface receptor Neuropilin-1, causing cell death, and that the overexpression of S1 can induce ferroptosis of SH-SY5Y [38]. Other studies have demonstrated the effects of S1 protein on mitochondrial functions [39], which may lead to cell death due to energy depletion.

The causes and particular cellular and molecular mechanisms of adverse effects of mRNA vaccines in the brain are still under-investigated. Preclinical studies on an animal model showed no changes in the brain after intramuscular injection of the SARS-CoV-2 mRNA vaccine [21]. In our study, we have chosen to investigate the effects of SARS-CoV-2 mRNA vaccines on primary neuronal–glial co-cultures. Such an approach has an advantage, as by directly applying the vaccines to CGCs, we excluded the involvement of peripheral inflammatory cytokines that may cross the blood–brain barrier and thus possibly contribute to the neuroinflammatory responses in the brain. The novel and important finding of the present study was that treatment of mixed neuronal–glial co-cultures with mRNA vaccines at singular 1–100 ng/mL doses or triple applications of a 6 ng/mL dose for 7 days was not toxic to neurons, suggesting that mRNA vaccines do not appear to directly affect neurons under the tested conditions. Nevertheless, both mRNA vaccines at 50–100 ng/mL concentrations and their repeated application at 6 ng/mL doses caused significant microglial proliferation. However, other markers of neuroinflammation, such as the secretion of proinflammatory cytokines, generation of reactive oxygen species, or the increased phagocytic activity of microglia, were not detected in cell co-cultures. Moreover, even stimulation of microglial proliferation by repeated doses of vaccines was only temporary and disappeared after a longer, 7-day period. All this suggests that mRNA vaccines induce only a mild and temporary inflammatory response in brain cell cultures, providing additional, indirect evidence for the safety of mRNA vaccines investigated.

In our study, we found that vaccines had a significant effect on microglial proliferation, which serves as one of the biomarkers of inflammatory response. We also evaluated additional markers of a potential inflammatory microglial response, including phagocytic activity, hydrogen peroxide production, and cytokine secretion. However, these were not changed under treatment with vaccines. Therefore, the increased number of microglia was statistically significant, but it was the only marker of microglial activation. It is likely that such temporary activation of microglia, without the production of reactive oxygen species and proinflammatory cytokines, does not lead to more severe, damaging effects on neuronal cells. This needs to be investigated in future research, particularly in in vivo models.

We also used a model of organotypic brain slice cultures, in which microglia can be observed under more physiological conditions than in isolated cell cultures in vitro [34]. As it has been reported previously [34], primary microglia in vitro differ from slice microglia in gene expression that can influence microglial responses. Treating murine hippocampal brain slices with repeated mRNA vaccine applications, we revealed different effects on male and female brain slices that have not been previously reported. We discovered that repeated T/R applications at 6 ng/mL increased microglial proliferation in female brain slice cultures but had no effect on male brain slices. Repeated application of O/OBA at 6 ng/mL had no effect on neuronal number, neither in female nor in male brain slices. These findings suggest that female brains might be more vulnerable to mRNA vaccines and exhibit a stronger inflammatory response. In addition, this points to the necessity to take into account gender differences in the studies on effectiveness as well as side effects of new vaccination means.

Our findings on possible sex differences in the response to SARS-CoV-2 mRNA vaccines are in line with recent clinical data. Several studies on the safety of the SARS-CoV-2 mRNA vaccines have found that women more often than men report side effects of vaccination, such as headaches, fatigue, and muscle pain [40]. The reasons for this are not entirely clear but may include biological factors such as a more active immune system, higher levels of antibodies in females versus males, influence of hormones, etc.

While our study provided new data on the effects of SARS-CoV-2 S1 protein and mRNA vaccines on neuronal viability and microglial activation, there were also some limitations. The in vitro studies presented in this work are more suitable for investigating the responses of particular cell types or molecular signaling pathways; however, responses may differ in more complex in vivo models. The second important limitation of this study is that we concentrated on cell death pathways and measured only a few inflammatory cytokines. It is possible that cytokines could be released in earlier stages of treatment, and the effect was simply off before taking the sample, or that prolonged treatment prevented the elevation in proinflammatory cytokines. However, it is now generally accepted that microglial activation may exhibit morphological, functional, and biochemical patterns in various pathological conditions and may be characterized by a variety of different biomarkers (therefore, activation is “disease-dependent”). In our study, we found that the application of mRNA vaccines resulted in certain microglial activation expressed as increased proliferation but without the secretion of inflammatory cytokines or ROS. Therefore, this kind of microglial activation seems to be mild, without a damaging effect on neurons, and is temporary, as it was found to last only a few days. In future research, it would be important to investigate a wide range of biomarkers of microglial activation and to determine the microglial phenotype (inflammatory or neuroprotective) that can occur after treatment with mRNA vaccines.

## 5. Conclusions

In summary, our results show that T/R and O/OBA mRNA vaccines induce short-term stimulation of microglial proliferation without other inflammatory signs, but they did not reduce neuronal viability in primary neuronal–glial co-cultures over time, even though the exposure to the external SARS-CoV-2 S1 protein may reduce neuronal viability. SARS-CoV-2 vaccines exert a stronger effect on female brain cells.

## Data Availability

All original data presented in this study are included in the article.

## Supplementary Materials

The following supporting information can be downloaded at https://www.mdpi.com/article/10.3390/medicina62010198/s1, Figure S1: The effects of SARS-CoV-2 mRNA vaccines on neuronal viability and cell count in CGC cultures after 3 and 7 days of treatment. (a) T/R effect on neuronal viability; (b) O/OBA effect on neuronal viability; (c) T/R effect on neuronal number; (d) O/OBA effect on neuronal number. Neuronal viability was presented as a percentage of live neurons from the total number of neurons in each group. Neuronal cell number expressed as a percentage of the number of neurons in the respective control group (0 ng/mL); Figure S2: The effects of repeated treatment with SARS-CoV-2 mRNA T/R and O/OBA vaccines on CGC cultures after 7-day incubation. (a) Neuronal viability presented as percentage of live neurons from the total number of neurons in each group; (b) Neuronal cell number expressed as a percentage of the number of neurons in the respective control group.

## Abbreviations

The following abbreviations are used in this manuscript:

ACE2	Angiotensin Converting Enzyme–2
CGC	Cerebral Granular Cells
GFP	Green Fluorescent Protein
HBSS	Hanks’ Balanced Salt Solution
IL-6	Interleukin 6
IL-1β	Interleukin 1 beta
iNOS	Inducible Nitric Oxide Synthase
mRNA	Messenger Ribonucleic Acid
NF-κB	Nuclear Factor Kappa B
O/OBA	Original/Omicron BA.4-5 COVID-19 mRNA Vaccine
RFP	Red Fluorescent Protein
ROS	Reactive Oxygen Species
SARS	Severe Acute Respiratory Syndrome
SARS-CoV-2 S1	SARS-CoV-2 Spike protein 1 subunit
SEM	Standard Error of the Mean
SPIM/LSFM	Selective Plane Illumination Microscopy/ Light Sheet Fluorescence Microscopy
T/R	Tozinameran/Riltozinameran COVID-19 mRNA Vaccine
