# Supplementary material for: Effects of Exogenous SARS-CoV-2 S1 Protein and mRNA Vaccines on Mixed Neuronal–Glial Cell Cultures
Source: Medicina (Kaunas). 2026 Jan 17;62(1):198. doi: 10.3390/medicina62010198 (PMC12843422; doi:10.3390/medicina62010198)
Supplement: Supplementary file 1 [file medicina-62-00198-s001.zip › Supplementary Figures.pdf]

medicina

MDPI

Figure S1

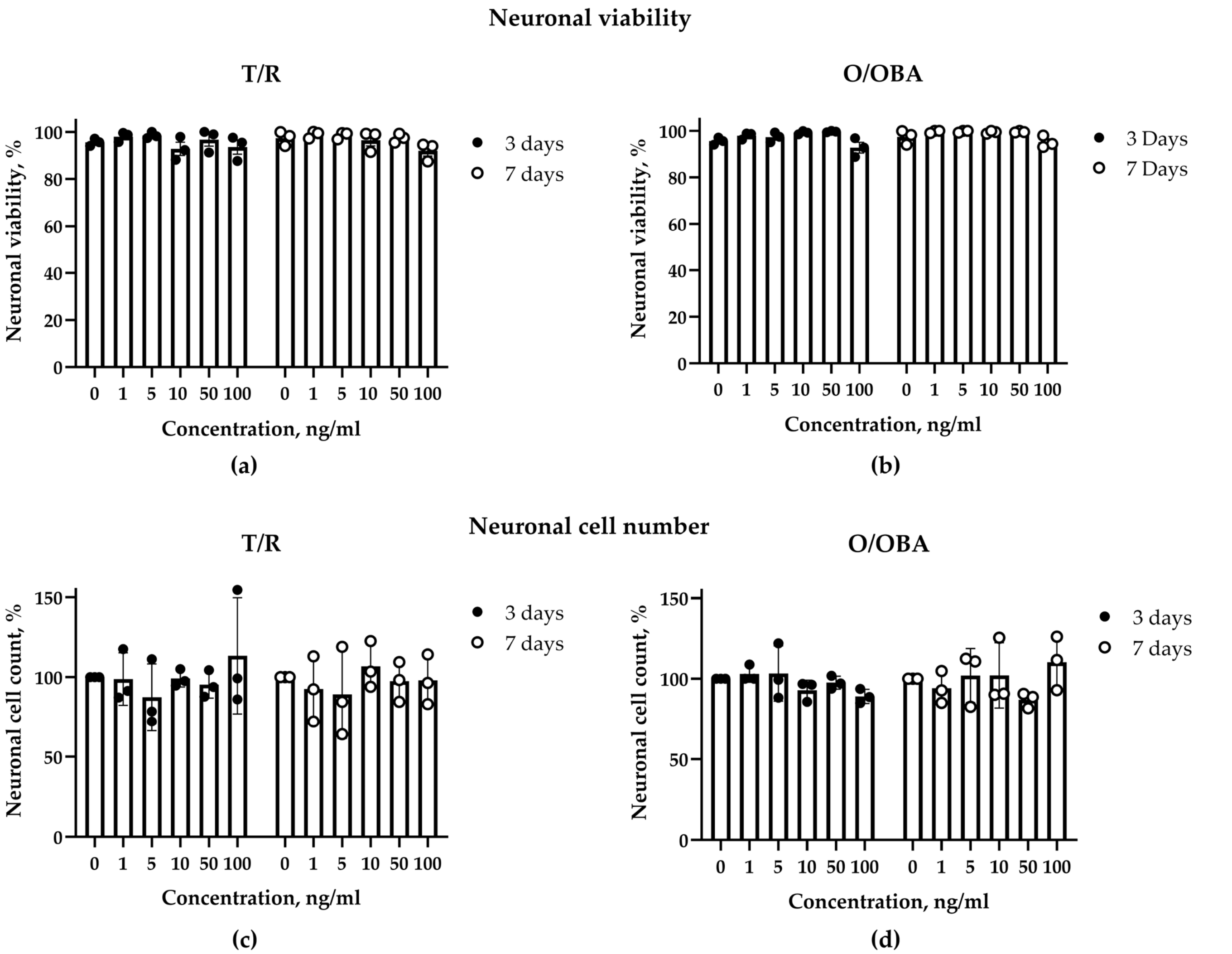

Figure S1 The effects of SARS-CoV-2 mRNA vaccines on neuronal viability and cell count in CGC cultures after 3 and 7 days of treatment. **(a)** T/R effect on neuronal viability; **(b)** O/OBA effect on neuronal viability; **(c)** T/R effect on neuronal number; **(d)** O/OBA effect on neuronal number. Neuronal viability was presented as a percentage of live neurons from the total number of neurons in each group. Neuronal cell number expressed as a percentage of the number of neurons in the respective control group (0 ng/mL).

Figure S2

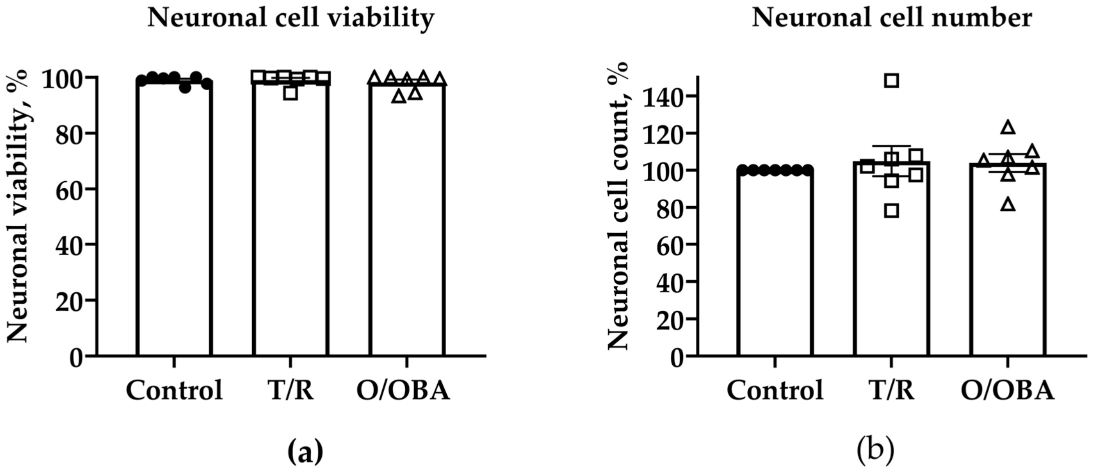

Figure S2 The effects of repeated treatment with SARS-CoV-2 mRNA T/R and O/OBA vaccines on CGC cultures after 7-day incubation. (**a**) Neuronal viability presented as percentage of live neurons from the total number of neurons in each group; **(b)** Neuronal cell number expressed as a percentage of the number of neurons in the respective control group.

https://doi.org/10.3390/medicina62010198
